# GC/MS Analyses of the Essential Oils Obtained from Different *Jatropha* Species, Their Discrimination Using Chemometric Analysis and Assessment of Their Antibacterial and Anti-Biofilm Activities

**DOI:** 10.3390/plants11091268

**Published:** 2022-05-09

**Authors:** Mariam I. Gamal El-Din, Fadia S. Youssef, Ahmed E. Altyar, Mohamed L. Ashour

**Affiliations:** 1 Department of Pharmacognosy, Faculty of Pharmacy, Ain-Shams University, Abbasia, Cairo 11566, Egypt; dr_mariam_gamal_eldin@pharma.asu.edu.eg (M.I.G.E.-D.); fadiayoussef@pharma.asu.edu.eg (F.S.Y.); 2 Department of Pharmacy Practice, Faculty of Pharmacy, King Abdulaziz University, P.O. Box 80260, Jeddah 21589, Saudi Arabia; aealtyar@kau.edu.sa; 3 Department of Pharmaceutical Sciences, Pharmacy Program, Batterjee Medical College, P.O. Box 6231, Jeddah 21442, Saudi Arabia

**Keywords:** antibacterial, antibiofilm, chemometrics, essential oils, euphorbiaceae, GC/MS, *Jatropha*, molecular docking, sustainability of natural resources, drug discovery

## Abstract

The essential oils of *Jatropha intigrimma*, *J. roseae* and *J. gossypifolia* (Euphorbiaceae) were analyzed employing GC/MS (Gas Chromatography coupled with Mass Spectrometry) analyses. A total of 95 volatile constituents were identified from *J. intigrimma, J. gossypifolia* and *J. roseae* essential oils, accounting for 91.61, 90.12, and 86.24%, respectively. Chemometric analysis using principal component analysis (PCA) based on the obtained GC data revealed the formation of three discriminant clusters due to the placement of the three *Jatropha* species in three different quadrants, highlighting the dissimilarity between them. Heneicosane, phytol, nonacosane, silphiperfol-6-ene, copaborneol, hexatriacontane, octadecamethyl-cyclononasiloxane, 9,12,15-Octadecatrienoic acid, methyl ester and methyl linoleate constitute the key markers for their differentiation. *In vitro* antibacterial activities of the essential oils were investigated at doses of 10 mg/mL against the Gram-negative anaerobe *Escherichia coli* using the agar well diffusion method and broth microdilution test. *J. gossypifolia* essential oil showed the most potent antimicrobial activity, demonstrating the largest inhibition zone (11.90 mm) and the least minimum inhibitory concentration (2.50 mg/mL), followed by the essential oil of *J. intigrimma*. The essential oils were evaluated for their anti-adhesion properties against the Gram-negative *E. coli* biofilm using a modified method of biofilm inhibition spectrophotometric assay. *J. intigrimma* essential oil showed the most potent biofilm inhibitory activity, demonstrating the least minimum biofilm inhibitory concentration (MBIC) of 31.25 µg/mL. *In silico* molecular docking performed within the active center of *E. coli* adhesion protein FimH showed that heneicosane, followed by cubebol and methyl linoleate, displayed the best fitting score. Thus, it can be concluded that the essential oils of *J. gossypifolia* and *J. intigrimma* leaves represent promising sources for antibacterial drugs with antibiofilm potential.

## 1. Introduction

Essential oils are natural, volatile components with a complex nature, possessing mostly fragrant odors manufactured by the plants as secondary metabolites. They are commonly prepared either by hydro-distillation or steam distillation; meanwhile, they are highly popular due to their observable biological potential, which is highly attributed to their different classes of compounds, particularly terpenoids. They are popular for their antimicrobial, antiviral, anti-inflammatory, analgesic, spasmolytic, anticancer, anti-aging and anesthetic activities, in addition to their wide consumption in the preservation of foods [1,2,3,4,5].

Microorganisms, a severe hazard attacking human beings, are characterized by the formation of an architectural colony inside an extracellular matrix of polymeric substances termed a biofilm. Bacterial biofilms are highly pathogenic and can trigger nosocomial infections [6,7]. It is worth highlighting that the National Institutes of Health (NIH) declared that 65% of microbial and 80% of chronic infections are accompanied by biofilm formation, which in turn occurs through many steps [4]. These steps comprise the attachment of the bacteria with living or non-living surfaces that are consequently followed by the production of a micro-colony that in turn forms three-dimensional structures and ends up, after maturation, with detachment [8]. Bacterial biofilms are highly contributed to the pronounced resistance of bacteria toward both antibiotics as well as the human immune system and thus prohibition of biofilm formation is highly adopted as a successful strategy combating microbial infections and antibiotic resistance [9]. Hence, searching for effective anti-biofilm agents, particularly from natural origin, has become mandatory worldwide.

*Jatropha* is a genus of flowering plants belonging to the Euphorbiaceae that includes approximately 175 succulent plants, shrubs and trees. *Jatropha* is an extensively strong and economical plant genus that natively grows in tropical and subtropical regions propagating on wasteland. Different species of the genus have been reported for their antimicrobial activities, as well as their richness in diterpenes [10]. *J. intigrimma*, *J. roseae* and *J. gossypifolia* are three species that belong to the genus *Jatropha.* The essential oils of *J. intigrimma* and *J. gossypifolia* leaves were previously analyzed for their chemical composition. In addition, *J. intigrimma* and *J. gossypifolia* leaf oils were reported to possess strong antimicrobial activities *versus Bacillus cereus* and *Staphylococcus aureus* for the former and against *Escherichia coli*, *Enterococcus faecium,* and *Staphylococcus aureus* for the latter [11,12]. 

Thus, herein a comparative study was performed for the first time on the leaves of *J. intigrimma*, *J. roseae* and *J. gossypifolia* essential oils that were qualitatively and quantitatively examined for their volatile constituents employing GC/MS (Gas Chromatography coupled with Mass Spectrometry analyses. The volatile oil yield, the major volatile constituents and their percent in each of the examined oils were estimated. This was consequently followed by their discrimination using chemometric analysis to easily detect the differences among the different species, which undoubtedly reflects the variation in their biological behavior. The different essential oils were investigated for their antibacterial activities against the Gram-negative anaerobe *Escherichia coli*. In addition, their inhibitory activities against *E. coli* biofilm formation were assessed for the first time, as this represents the major cause of gastroenteritis, urinary tract infections and neonatal meningitis. The major compounds identified in the bioactive essential oil were further subjected to *in silico* studies to confirm the obtained results. Thus, herein we aimed to find new antimicrobial agents of natural origin with anti-biofilm potential that could be incorporated in pharmaceutical dosage form applied topically to eliminate microbial infection.

## 2. Results

### 2.1. Chemical Composition of the Essential Oils of J. intigrimma, J. gossypifolia and J. roseae Leaves

**Figure 1:**
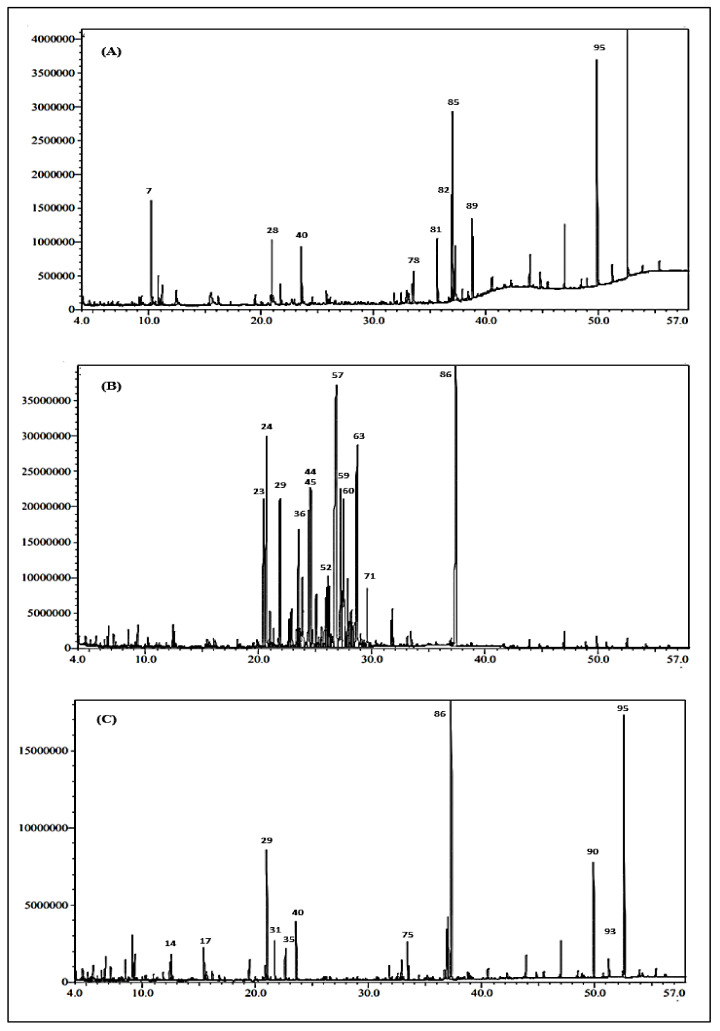
GC-chromatograms of the essential oils obtained from (**A**): *J. intigrimma,* (**B**): *J. gossypifolia* and (**C**): *J. roseae* leaves using the Rtx-5MS column.

A comparative investigation of the volatile constituents of the three *Jatropha* species, namely *J. intigrimma* Jacq., *J. gossypifolia* L. and *J. roseae* Radcl.-Sm., was conducted for the first time in the present study. The chemical compositions of the essential oils of the fresh leaves of the three species were qualitatively and quantitatively investigated by GC-MS (Figure 1) and compared with the previous results obtained by investigating the essential oils of *J. intigrimma* and *J. gossypifolia* grown in Nigeria. The yields of *J. intigrimma, J. gossypifolia* and *J. roseae* essential oils were estimated as 0.31 ± 0.11, 0.21 ± 0.09, and 0.19 ± 0.11% (*v*/*w*), respectively. A total of 95 volatile constituents were identified from the GC/MS analyses of *J. intigrimma, J. gossypifolia* and *J. roseae* essential oils, accounting for 91.61, 90.12, and 86.24% of their total oil content, respectively. A list of the identified volatile constituents, the percentage of each volatile component, their experimental retention indices and the literature retention indices, in an order of increasing retention indices (RIs) on the Rtx-5MS column, are summarized in Table 1.

Twenty-two volatile constituents were identified in the oil of *J. intigrimma* leaves, in which fatty acid esters represented the most prevailing class, constituting 22.26% of the oil constituents. In *J. intigrimma* oil, 9,12,15-octadecatrienoic acid methyl ester (10.77%), methyl linoleate (5.65%), and hexadecanoic acid methyl ester (3.14) were the most abundant fatty acid esters identified. Furthermore, hexatriacontane, octadecamethyl cyclononasiloxane, D-limonene, phytol and *β-*ionone were present in *J. intigrimma* oil in considerable quantities, representing 28.44, 8.42, 5.35, 3.85 and 3.53%, respectively.

Concerning *J*. *gossypifolia* oil, 61 volatile components were identified in which copaborneol, phytol and eudesma-4(15), 7-dien-1*β*-ol constituted the major volatile constituents of the oil, representing 15.70, 10.33 and 7.01% of the oil content, respectively. It is worth mentioning that sesquiterpene hydrocarbons and oxygenated sesquiterpenes are the predominant classes in *J. gossypifolia* oil, accounting for 74.86% of the oil content. Isosativene (4.08%), α-copaene (5.87%), spathulenol (3.63%), muurola-4,10(14)-dien-1β-ol (4.62%) and caryophylla-4(12),8(13)-dien-5α-ol (4.82%) were the most abundant sesquiterpenes present.

**Figure 2:**
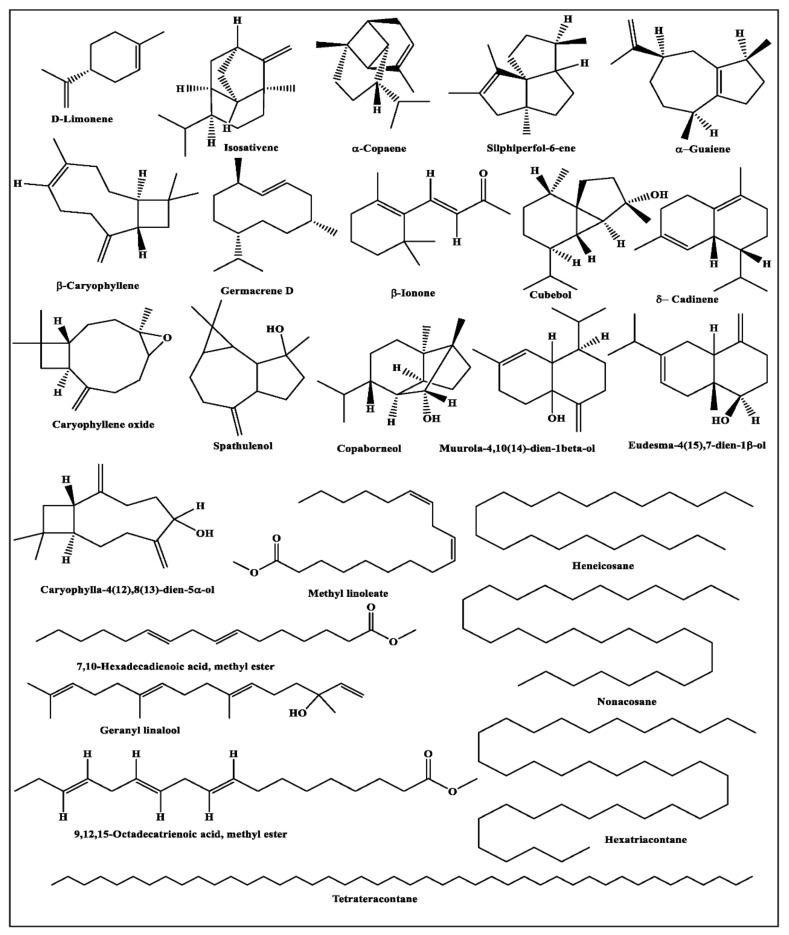
Major components identified in the essential oils obtained from *J. intigrimma, J. gossypifolia* and *J. roseae* leaves.

Meanwhile, 44 constituents were identified in *J. roseae* oil, where phytol, hexatriacontane and heneicosane constituted the major volatile constituents, representing 15.25, 14.50 and 12.67% of *J. roseae* oil, respectively. It is noteworthy that *J. roseae* oil is rich in diterpenes and higher alkanes, accounting for 63.48 % of the oil. However, different sesquiterpene hydrocarbons, oxygenated sesquiterpenes and fatty acids esters can also be observed in *J. roseae* oil viz. silphiperfol-6-ene (6.90%), *β-*ionone (3.09%), *α*-guaiene (2.00%), 7,10-hexadecadienoic acid, methyl ester (1.36%), 9,12-octadecadienoic acid, methyl ester (2.47%) and 9,12,15-octadecatrienoic acid, methyl ester (3.18%). A scheme representing the major compounds present in the three *Jatropha* species is presented in Figure 2.

### 2.2. Discrimination of the Three Jatropha Species Using GC Data Coupled with Chemometrics

**Figure 3:**
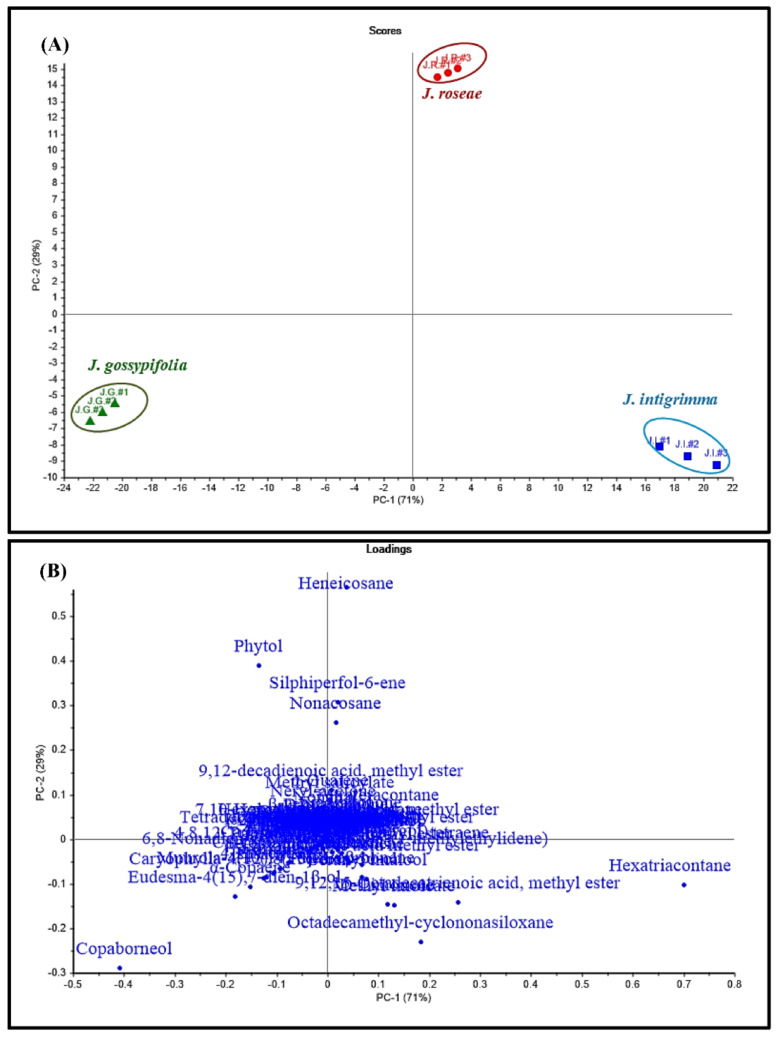
Score plot (**A**) and loading plot (**B**) of GC data collected from *J. intigrimma*, *J. gossypifolia* and *J. roseae* leaves essential oil analyses using unsupervised chemometric analysis (PCA).

Chemometric analysis was adopted using an unsupervised pattern recognition technique represented by principal component analysis (PCA) based on the obtained GC data. Chemometric analysis constitutes an advanced approach for the better discrimination of closely related species, relying upon data gathered from different chromatographic and spectroscopic techniques. Principal component analysis (PCA) was initially performed to categorize data and to correlate between the examined samples and the used variables [13]. PCA based upon the number as well as the relative peak area of volatile constituents obtained from GC spectra for different *Jatropha* species, illustrated in Figure 3, revealed the formation of three discriminant clusters representing the three species. 

PCA score plot for principal components (PCs), which are PC1 versus PC2, illustrated in Figure 3A accounts for 71% and 29% of the total variance, respectively. This perfectly results in the placement of the three *Jatropha* species in three different quadrants, which in turn highlights the evident dissimilarity between the three species. Both PC1 and PC2 effectively discriminate between *J. gossypifolia* and *J. roseae,* where the former lies in the left lower quadrant showing negative values for both PCs in contrast to the latter that is positioned in the upper right quadrant revealing positive values for both PCs. Regarding *J. intigrimma* that lies in the right lower quadrant in the PCA score plot, only PC1 could effectively discriminate between it and *J. gossypifolia,* as *J. intigrimma* showed positive values, while *J. gossypifolia* showed negative values for PC1. However, *J. intigrimma* and *J. roseae* could be discriminated only via PC2, where the former displayed negative values and the latter showed positive values. By careful analysis of the loading plot illustrated in Figure 3B, it was clearly obvious that heneicosane, phytol, nonacosane and silphiperfol-6-ene were the key markers for the discrimination of *J. roseae* from the other two species, while copaborneol constitutes the key marker for its differentiation from the other two species. Regarding *J. intigrimma*, hexatriacontane, octadecamethyl-cyclononasiloxane*,* 9,12,15-Octadecatrienoic acid, methyl ester and methyl linoleate represent the key markers. The results from chemometric analysis coupled with GC data allowed the clustering of samples, and this undoubtedly leads to better visualization of the differences among the essential oils obtained from different *Jatropha* species and in turn reflected the differences between their biological behaviors.

### 2.3. Evaluation of Antibacterial and Anti-Biofilm Activity

#### 2.3.1. Antibacterial Activities of *J. intigrimma*, *J. gossypifolia* and *J. roseae* Essential Oils

*In vitro* antibacterial activities of the essential oils obtained from the leaves of the three *Jatropha* species, *J. intigrimma*, * J. gossypifolia* and *J. roseae*, were investigated at doses of 10 mg/mL against the Gram-negative anaerobe *Escherichia coli*. The agar well diffusion method was adopted for calculating the mean diameter of inhibition zones produced by the three oil samples in comparison with the standard antimicrobial drug, Gentamicin. Furthermore, the minimum inhibition concentrations (MIC) values were estimated for the oil samples using the broth microdilution test. The essential oil obtained from *J. gossypifolia* leaves demonstrated the most potent antimicrobial activity against *E. coli*, demonstrating the largest inhibition zone (11.90 ± 0.46 mm) and the least minimum inhibitory concentration (2.50 mg/mL), followed by the essential oil obtained from *J. intigrimma* leaves. The latter exhibited an inhibition zone of 9.57 ± 0.40 mm and an MIC of 5.00 mg/mL. The oil of *J. roseae* demonstrated the least inhibition zone (8.93 ± 0.60 mm) but had MIC values equal to those of *J. intigrimma* oil of 5.00 mg/mL. The results were compared to the standard gentamycin that exhibited a mean inhibition zone of 27.09 ± 0.01 mm at a dose of 4 µg/mL and demonstrated an MIC of 2 µg/mL. This experiment was repeated in triplicate and data were represented as mean ± S.D.

#### 2.3.2. Antibiofilm Activities of *J. intigrimma*, *J. gossypifolia* and *J. roseae* Essential Oils

The essential oils of the different *Jatropha* species were further evaluated for their potential anti-adhesion properties against the Gram-negative *E. coli* biofilm. A modified method of biofilm inhibition spectrophotometric assay was adopted for the determination of the inhibitory activity of essential oils against the formation of *E. coli* biofilm and for the calculation of the minimum concentration required for complete inhibition of visible biofilm cell growth (MBIC). The essential oil of *J. intigrimma* demonstrated the most potent biofilm inhibitory activity, demonstrating the least minimum biofilm inhibitory concentration (MBIC) of 31.25 µg/mL. However, the essential oils of *J. roseae* and *J. gossypifolia* demonstrated less potent antibiofilm activities, demonstrating minimum biofilm inhibitory activities (MBIC) of 250 and above 1000 µg/mL, respectively, as displayed in Table 2.

### 2.4. Molecular Docking Studies of Adhesion Proteins with Major Constituents in Jatropha Essential Oils

**Figure 4:**
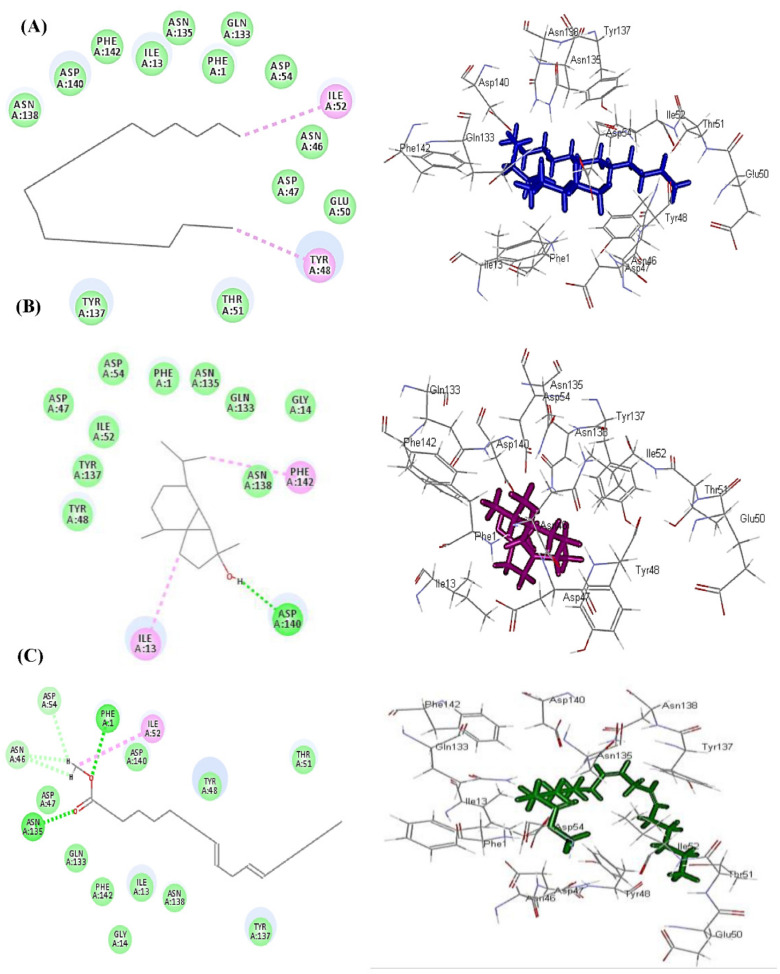
2D and 3D binding modes of heneicosane (**A**), cubebol (**B**) and methyl linoleate (**C**) within the active center of *E. coli* adhesion protein FimH using *in silico* studies employing the C-docker protocol.

*In silico* molecular docking of the major compounds identified from *Jatropha* essential oils was performed within the active site of the adhesion proteins associated with *E. coli* that enable the bacterium to attach to the surfaces and consequently form its invasive biofilm as FimH (PDB ID 1TR7; 2.10 Å) downloaded from the protein data bank. The docking experiments were carried out using Discovery Studio 4.5 (Accelrys Inc., San Diego, CA, USA) using the C-Docker protocol. The results displayed in Table 3 revealed that heneicosane, followed by cubebol and methyl linoleate, displayed the best fitting score within the active center of *E. coli* adhesion protein FimH with free binding energies equal to −30.68, −8.92 and −4.55 Kcal/mole, respectively. Heneicosane forms two alkyl and π-alkyl bonds with Ile52 and Tyr48, in addition to the formation of Van der Waals interactions with many amino acid residues at the active center (Figure 4A). However, cubebol forms one conventional H-bond with Asp140, in addition to two alkyl and π-alkyl bonds with Ile13 and Phe142 together with Van der Waals bonds at the active site (Figure 4B). Regarding methyl linoleate, it forms two conventional H-bonds with Asn135 and Phe1, one alkyl bond with Ile52, in addition to three C-H bonds with Asp54 and Asn46, together with many Van der Waals interactions, as shown in Figure 4C.

## 3. Discussion

This study represents the first report investigating the volatile constituents of *J. rosea* leaf oil. In addition, a comparative investigation of the volatile constituents of the three *Jatropha* species, *J. intigrimma*, *J. gossypifolia* and *J. roseae*, was conducted. A previous study on *J. intigrimma* leaves obtained from Nigeria reported *β-*ionone as one of the major volatile constituents of the oil, which was also identified in our study in a lower concentration. However, the other major constituents reported by Eshilokun et al., pentadecanal and 1,8-cineole, were absent in the present study [12]. Previous literature by Aboaba et al. on *J. gossypifolia* leaves grown in Nigeria reported the predominance of sesquiterpenes accounting for 74.3% of the oil content, which is almost relative to our study [14]. Meanwhile, fatty acids were reported by Ababa et al. in *J. gossypifolia* oil despite their scarcity in our *J. gossypifolia* oil. The major constituents reported by Aboaba et al., germacrene and hexahydrofarnesyl acetone, were identified in the present study but in negligible quantities. Another study in different regions of Nigeria [11] reported the predominance of phytol (33.4%) and linalool (9.81%) in *J. gossypifolia* oil, which were identified in the current study in different percentages.

The chemical composition variability among the essential oils of *Jatropha* species grown in Egypt and those in different regions of Nigeria or elsewhere are attributable to multiple exogenous and endogenous factors, such as seasonal variation, geographical region affecting soil, precipitation and light exposure, extraction method, and the age of the plant and its different chemotypes [15]. Additionally, chemometric analysis was adopted using an unsupervised pattern recognition technique represented by principal component analysis (PCA) based on the obtained GC data. PCA based upon the number, as well as the relative peak area, of volatile constituents obtained from GC spectra for different *Jatropha* species revealed the formation of three discriminant clusters due to the placement of the three *Jatropha* species in three different quadrants, which in turn highlights the evident dissimilarity between the three species evidenced by the score plot. By careful analysis of the loading plot, it was obvious that heneicosane, phytol, nonacosane and silphiperfol-6-ene, copaborneol, hexatriacontane, octadecamethyl-cyclononasiloxane, 9,12,15-Octadecatrienoic acid, methyl ester and methyl linoleate constituted the key markers for the differentiation of the three species.

Essential oils have long been known for their antimicrobial potential that made them crucial in different fields, including the food industry, preservation and medication [16]. To the best of our knowledge, few reports have addressed the antimicrobial activities of essential oils of different *Jatropha* species. Our current study presented the first report on the antimicrobial activities of *J. intigrimma* and *J. roseae* essential oils against *E. coli* Gram-negative bacterium and compared them with the activity of *J. gossypifolia* essential oil. The results of the *in vitro* antibacterial activities of the essential oils exhibited the superior potency of *J. gossypifolia* essential oil, followed by *J. intigrimma* essential oil, against the Gram-negative anaerobe *E. coli*. Results were in accordance with the previous literature reporting the bacteriostatic activity of *J. gossypifolia* leaf oil against the Gram-negative bacterium *E. coli* at a dose of 0.10 mg/mL [11]. It is worth mentioning that phytol, a major constituent of *J. gossypifolia* leaf oil constituting 10.33 % of the oil content, was previously reported for its potent antimicrobial activity against *Escherichia coli*, exhibiting growth inhibition at a minimum concentration (MIC) of 62.5 μg/mL [17,18]. Moreover, caryophylline oxide, identified in *J. gossypifolia* leaf oil, was reported to possess moderate inhibitory activity against Gram-negative *E. coli* with an estimated MIC of 60 ppm [19]. 

Bacterial biofilms are colonies of microorganisms lying in a matrix of polysaccharides attached to surfaces. They represent physical barriers that inhibit the penetration of antimicrobials to their target sites. Hence, bacterial biofilms represent rational biological risks in drinking water, food, and clinical and industrial environments [20]. Nowadays, increased interest has been directed toward investigating the different mechanisms of inhibiting bacterial biofilm formation and growth. Because attachment represents the initial step in almost all types of biofilm formation, the antiadhesive properties of natural products have recently become a prime interest of study in an aim for the early prevention of microbial biofilm and inhibiting the formation of micro colonies [21]. Furthermore, inhibiting the cell attachment of microbial biofilms has been found to be more readily achieved than preventing the growth of already established biofilms [22]. Hence, the three *Jatropha* oils were investigated for their inhibitory activities against *E. coli* adhesion. This study represents the first report of the antibiofilm activities of the essential oils of the three *Jatropha* species. The results demonstrated the superior antibiofilm activity of the essential oil of *J. intigrimma* leaves compared to the other two *Jatropha* oils. In addition, *in silico* molecular docking of the major compounds identified from *Jatropha* essential oils was performed within the active site of one of the adhesion proteins associated with *E. coli* that enables the bacterium to attach to the surfaces and consequently forms its invasive biofilm, FimH. The docking experiments revealed that heneicosane, followed by cubebol and methyl linoleate, displayed the best fitting score within the active center of *E. coli* adhesion protein FimH with free binding energies equal to −30.68, −8.92 and −4.55 Kcal/mole, respectively.

Previous literature has reported the anti-biofilm activities of different fatty acids and their methyl esters [23,24]. In addition, the monoterpene D-Limonene, an essential component of *J. intigrimma* essential oil, has been reported to possess strong anti-biofilm activity against the heterotrophic, Gram-negative, rod-shaped bacterium *Aeromonas hydrophila* isolated from fish [25]. The anti-adhesive properties and the capabilities of inhibiting the initial biofilm formation can be explained by the possibility of interference with the attraction forces that support the bacterial film with the affected surface or by interrupting the access of vital nutrients for bacterial growth and adhesion [26]. Thus, the antibiofilm of the essential oil could be attributed to the synergistic action of the existing compounds.

## 4. Materials and Methods

### 4.1. Plant Material

The fresh leaves of the three *Jatropha* species: *J. intigrimma* Jacq., *J. gossypifolia* L. and *J. roseae* Radcl.-Sm. (Euphorbiaceae) were collected from plants grown in Mazhar Botanical Garden, Giza, Egypt, on August 2020. The plants were kindly identified and authenticated by Eng. Terase Labib, Consultant of Plant Taxonomy at the Ministry of Agriculture and El-Orman Botanical Garden, Giza, Egypt. Voucher specimens of the authenticated plant with codes BMC-JI-MLA, BMC-JG-MLA and BMC-JR-MLA were kept at the Department of Pharmaceutical Sciences, Pharmacy Program, Batterjee Medical College.

### 4.2. Chemicals, Reagents and Strains

Dimethyl sulfoxide (DMSO), crystal violet and trypan blue dye were obtained from Sigma (St. Louis, MO, USA). Crystal violet stain was prepared as 1% using 0.5% (*w/v*) crystal violet and 50% methanol that were adjusted to volume using distilled water and subsequently filtered through a Whatman No.1 filter paper. The studied bacterial strain was *Escherichia coli,* ATCC 25922, obtained from the American type culture collection (ATCC).

### 4.3. Preparation of Essential Oils

The fresh leaves of the three *Jatropha* species were separately hydro-distillated for 6 h utilizing a Clevenger-type apparatus. The obtained essential oils were dried over anhydrous sodium sulfate and gathered in separate and sealed vials that were maintained at −30 °C until further analyses. The yield was calculated as % *v/w* after being determined in triplicate, where calculation was performed based on the initial plant weight.

### 4.4. Metabolic Profiling of the Essential Oils Obtained from J. intigrimma, J. gossypifolia and J. roseae Using GC/MS Analysis

Gas chromatography coupled with Mass Spectrometry (GC/MS) analyses were done on Shimadzu GCMS-QP 2010 (Shimadzu Corporation, Koyoto, Japan) accompanied by Rtx-5MS (30 m × 0.25 mm i.d. × 0.25 µm film thickness) capillary column (Restek, PA, USA) and attached to a Shimadzu mass spectrometer. An initial set of temperature of the column at 50 °C for 3 min was done that was gradually elevated from 50 °C to 300 °C at a rate of 5 °C/min, followed by isothermal maintenance for 10 min at 300 °C. The injector temperature was maintained at 280 °C, while the interface and the ion source temperature were kept at 220 and 280 °C, respectively. The flow rate of helium, which was used as a carrier gas, was 1.37 mL/min. One microliter was injected from the diluted sample with a concentration of 1% *v/v* through a split mode using a split ratio of 15:1. The mass spectrum was recorded using an EI mode of 70 eV in the range of *m/z* 35 to 500. Compound quantitation relied upon the normalization method, taking the reading of three chromatographic runs. Identification of compounds was achieved depending on the retention indices of the detected compounds with regard to a homologous series of *n*-alkanes (C8–C28) that were injected under the same conditions and via comparison mass spectra of the detected compounds with those recorded in the Wiley library database as well as the National Institute of Standards and Technology (NIST) and together with the literature [1,27,28,29]. 

### 4.5. Discrimination of the Three Jatropha Species Using GC Data Coupled with Chemometrics

Chemometric analysis using principal component analysis (PCA) as an unsupervised pattern recognition technique was done based on the obtained GC using CAMO’s Unscrambler^®^ X 10.4 software (Computer-Aided Modeling, As, Norway) as previously described [3,5]. This was done in an effort to allow the clustering of samples, which undoubtedly leads to better visualization of the differences among the essential oils obtained from different *Jatropha* species.

### 4.6. Evaluation of Antibacterial and Anti-Biofilm Activity

#### 4.6.1. Susceptibility Test Using the Agar Well Diffusion Method

Susceptibility tests were performed according to NCCLS recommendations (National Committee for Clinical Laboratory Standards) [30]. Screening tests concerning the inhibition zone were performed employing the well diffusion assay previously conducted by Hindler et al. [31]. Preparation of the inoculum suspension was performed from cultures grown overnight on an agar plate that were concomitantly inoculated into Mueller–Hinton broth. A sterile swab was adopted for the inoculation of Mueller–Hinton agar plates (fungi using malt agar plates) after being immersed in the inoculum suspension. The examined samples at different concentrations (2.5, 5 and 10 mg/mL) were solubilized in dimethyl sulfoxide (DMSO) and the inhibition zones were determined around each well after 24 h at 37 °C where the control was prepared using DMSO.

#### 4.6.2. Determination of the Minimum Inhibitory Concentration (MIC) Using the Broth Microdilution Method

The minimum inhibitory concentration (MIC) was determined as previously recommended by the Clinical and Laboratory Standards Institute (CLSI). Briefly, the dilution of a stock solution composed of 10% of the examined oil in the brain heart infusion broth (BHI) in two-fold serial dilutions was performed to obtain 0.02 to 25 mg/mL concentrations at a total volume of 100 mL per well in 96-well microtiter plates. One-hundred milliliters of each tested strain adopting a concentration of 1 × 106 CFU/mL were added to each well, followed by their incubation at 37 °C in appropriate conditions. The medium was used as the non-treated control, while 10% DMSO was employed as the negative control, whereas 0.1% (*w/v*) CHX was the positive control. MIC is the lowest concentration that completely prohibited growth when compared to the non-treated control. All experiments were repeated three times in duplicate.

#### 4.6.3. Evaluation of Anti-Biofilm Activity 

The volatile oil samples obtained from hydro-distillation of the three *Jatropha* species were evaluated for their inhibitory activity against biofilm formation of the Gram-negative anaerobe *Escherichia coli*. Biofilm inhibition assay was performed in 96-well plates adopting the modified method of biofilm inhibition spectrophotometric assay [32]. Briefly, 100 μL of an *Escherichia coli* cell suspension was added to a 96-well titer plate together with different concentrations of samples (1000, 500, 250, 125, 62.5, 31.25, 15.63 and 7.81 μg/mL); in addition, DMSO was added and incubated for 24 h at 37 °C. After incubation, the liquid suspension was removed, and 100 μL of 1% *w/v* aqueous solution of crystal violet was added. Removal of the excess crystal violet was achieved after 30 min of staining at room temperature followed by washing the wells thoroughly and the addition of 95% ethanol and incubation for 15 min. The reaction mixture was read spectrophotometrically at a wavelength of 570 nm using a microplate reader (TECAN, Inc.) after being shaken gently. The percent of inhibition of biofilm formation was determined according to the following equation:

% inhibition = OD in control − OD in treatment × 100 OD in control.

The relation between biofilm formation inhibitory % and drug concentration is plotted to obtain the inhibitory curve after treatment with the specified compound. MBIC was the concentration required to completely inhibit biofilm formation.

### 4.7. Molecular Docking Studies of Adhesion Proteins with Major Constituents in Jatropha Essential Oils

Molecular docking analysis was performed on the major constituents existing in *Jatropha* essential oils regarding adhesion proteins associated with *E. coli* that enable the bacterium to attach to the surfaces and consequently form its invasive biofilm as FimH (PDB ID 1TR7; 2.10 Å) [33]. This protein was downloaded from the protein data bank and docking experiments were carried out using Discovery Studio 4.5 (Accelrys Inc., San Diego, CA, USA) using the C-Docker protocol as previously reported [5,34,35,36], where binding energies (∆*G*) were calculated from the following equation:

Δ*G*_binding_ = E_complex_ − (E_protein_ + E _ligand_) Where;

Δ*G*_binding_: The ligand–protein interaction binding energy, 

E_complex_: The potential energy for the complex of protein bound with the ligand, 

E_protein:_ The potential energy of protein alone and

E_ligand_: The potential energy for the ligand alone.

## 5. Conclusions

In conclusion, the essential oil obtained from *J. intigrimma, J. gossypifolia* and *J. roseae* leaves revealed considerable variation, as revealed by GC analyses. This variation becomes clearly obvious when coupled with chemometric analysis that results in the placement of the three *Jatropha* species in three different quadrants, which in turn highlights the evident dissimilarity between the three species. Moreover, the essential oils of *J. gossypifolia* and *J. intigrimma* leaves represent promising sources for a new generation of antibacterial drugs. Their distinctive antibacterial and antibiofilm activities are probably attributed to their major bioactive chemical constituents, as well as the possible synergistic effect among them. To further confirm the obtained results, *in silico* molecular docking of the major compounds identified from *Jatropha* essential oils was performed within the active center of *E. coli* adhesion protein FimH and results showed that heneicosane followed by cubebol and methyl linoleate displayed the best fitting score. Thus, additional in vivo studies and bioavailability studies are highly recommended to ascertain the obtained results.

## Figures and Tables

**Table 1:** Essential oil compositions obtained from *J. intigrimma*, *J. gossypifolia* and *J. roseae* leaves using the Rtx-5MS column.

No.	Compounds ^[a]^	*RI*		Composition (%)			Identification
		Measured ^[b]^	Reported ^[c]^	*J. intigrimma*	*J*. *gossypifolia*	*J. roseae*	
1.	*n*-Nonane	880	900	-	0.20	0.78	MS, RI
2.	*α*-Pinene	915	915	-	0.08	-	MS, RI
3.	2-Methyl-nonane	947	951	-	0.05	0.15	MS, RI
4.	*β*-Pinene	987	982	-	0.30	-	MS, RI
5.	*trans*-2-(2-Pentenyl)furan	991	985	-	-	0.25	MS, RI
6.	*p*-Cymene	1014	1017	-	0.03	-	MS, RI
7.	D-Limonene	1018	1018	5.35	0.23	-	MS, RI
8.	(E)-*β*-Ocimene	1038	1038	1.90	0.09	-	MS, RI
9.	2,5-dimethyl-Nonane	1045	1042	-	0.04	0.09	MS, RI
10.	*γ*-Terpinene	1048	1048	1.76	0.07	-	MS, RI
11.	*p*-Cymenene	1069	1069	-	0.12	0.49	MS, RI
12.	α-Terpinolene	1088	1088	0.85	0.23	0.85	MS, RI
13.	*β*-Linalool	1090	1090	1.23	0.34	-	MS, RI
14.	Isophorone	1094	1094	-	0.24	1.23	MS, RI
15.	Nonanal	1098	1102	-	0.05	-	MS, RI
16.	1-Nonanol	1162	1159	-	0.03	0.09	MS, RI
17.	Methyl salicylate	1185	1187	-	-	1.87	MS, RI
18.	Safranal	1189	1189	1.90	-	-	MS, RI
19.	Decanal	1197	1195	-	0.10	0.48	MS, RI
20.	Cumaldehyde	1231	1230	-	-	0.42	MS, RI
21.	Carvacrol	1297	1298	-	0.01	0.08	MS, RI
22.	4-Vinylguaiacole	1311	1311	-	-	0.38	MS, RI
23.	*α*-Longipinene	1324	1327	0.64	-	-	MS, RI
24.	*α*-Cubebene	1340	1344	-	0.19	-	MS, RI
25.	Isosativene	1358	1359	-	4.08	-	MS, RI
26.	*α*-Copaene	1369	1369	-	5.87	-	MS, RI
27.	Longicyclene	1374	1374	-	-	0.74	MS, RI
28.	*β*-Bourbonene	1376	1376	3.14	0.17	-	MS, RI
29.	Silphiperfol-6-ene	1379	1380	-	-	6.90	MS, RI
30.	*Z-β*-Caryophyllene	1404	1407	1.55	-	-	MS, RI
31.	*α*-Guaiene	1404	1409	-	-	2.00	MS, RI
32.	*E-β*-Caryophyllene	1409	1409	-	2.97	0.06	MS, RI
33.	*α*-Ionone	1417	1421	-	-	0.07	MS, RI
34.	*β*-Ionone epoxide	1437	1430	-	0.14	-	MS, RI
35.	Neryl-acetone	1440	1445	-	0.48	1.65	MS, RI
36.	Humulene	1445	1445	-	0.60	-	MS, RI
37.	Alloaromadendrene	1453	1453	-	0.80	-	MS, RI
38.	Cadina-1(6),4-diene	1465	1469	-	0.21	-	MS, RI
39.	Germacrene D	1474	1474	-	2.09	-	MS, RI
40.	*β-*Ionone	1477	1478	3.53	0.39	3.09	MS, RI
41.	Cubebol	1488	1484	-	2.17	-	MS, RI
42.	*α*-Muurolene	1491	1491	-	0.37	-	MS, RI
43.	*β*-Himachalene	1500	1500	-	0.21	-	MS, RI
44.	δ-Cadinene	1507	1507	-	3.55	-	MS, RI
45.	δ-Guaijene	1524	1526	-	4.02	-	MS, RI
46.	Cubenol	1534	1538	-	0.91	-	MS, RI
47.	*α*-Calacorene	1544	1541	-	2.8	-	MS, RI
48.	*β-*Caryophyllene oxide	1556	1556	-	1.25	-	MS, RI
49.	4,8,12-Trimethyltrideca- 1,3,7,11-tetraene	1565	1565	0.84	0.40	-	MS, RI
50.	Globulol	1569	1568	-	0.95	-	MS, RI
51.	Spathulenol	1569	1569	-	3.63	-	MS, RI
52.	Pseudoionone	1575	1581	-	-	0.33	MS, RI
53.	Caryophyllene oxide	1578	1578	-	-	0.11	MS, RI
54.	Guaiol	1581	1584	-	-	0.19	MS, RI
55.	Humulene epoxide	1592	1592	-	0.33	-	MS, RI
56.	Davanone	1594	1592	-	-	0.28	MS, RI
57.	Copaborneol	1597	1593	-	15.7	-	MS, RI
58.	*1-epi-*Cubenol	1619	1617	tr.	-	tr.	MS, RI
59.	Muurola-4,10(14)-dien-1β-ol	1624	1630	-	4.62	-	MS, RI
60.	Caryophylla-4(12),8(13)-dien-5*α*-ol	1634	1640	-	4.82	-	MS, RI
61.	*α*-Cadinol	1650	1660	-	1.42	-	MS, RI
62.	Bulnesol	1664	1666	-	1.45	-	MS, RI
63.	Eudesma-4(15),7-dien-1*β*-ol	1680	1686	-	7.01	-	MS, RI
64.	Ylangenol	1698	1693	-	0.24	-	MS, RI
65.	(2E,6E)-Farnesol	1699	1695	-	-	0.22	MS, RI
66.	14-Hydroxy-*α*-humulene	1721	1718	-	0.13	0.07	MS, RI
67.	Z-ligustilide	1732	1741	-	0.13	-	MS, RI
68.	Benzyl benzoate	1758	1750	-	0.27	-	MS, RI
69.	3-Octadecene	1778	1784	-	-	0.21	MS, RI
70.	Tetradecanoic acid, 1-methylethyl ester	1805	1812	-	-	0.13	MS, RI
71.	Farnesyl acetate	1816	1818	-	1.06	-	MS, RI
72.	Hexahydrofarnesyl acetone	1825	1827	-	0.56	0.74	MS, RI
73.	Eudesmol acetate	1830	1830	-	tr.	-	MS, RI
74.	*n*-Hexadecan-1-ol	1861	1854	-	-	0.39	MS, RI
75.	7,10-Hexadecadienoic acid, methyl ester	1875	1894	1.50	-	1.36	MS, RI
76.	Palmitoleic acid, methyl ester	1886	1886	1.20	-	-	MS, RI
77.	Farnesyl acetone	1903	1897	-	tr.	-	MS, RI
78.	Hexadecanoic acid methyl ester	1906	1906	3.14	0.29	0.69	MS, RI
79.	1-Hexadecanol, acetate	1986	1978	-	-	0.28	MS, RI
80.	Octadecanal	1998	1999	-	0.08	0.31	MS, RI
81.	Geranyl linalool	2009	2002	3.36	-	-	MS, RI
82.	Sclareolide	2066	2065	-	0.01	0.85	MS, RI
83.	Methyl linoleate	2077	2076	5.65	-	-	MS, RI
84.	9,12-decadienoic acid, methyl ester	2077	2075	-	-	2.47	MS, RI
85.	9,12,15-Octadecatrienoic acid, methyl ester	2085	2085	10.77	-	3.18	MS, RI
86.	Phytol	2096	2096	3.85	10.33	15.25	MS, RI
87.	Verrucarol	2132	2025	-	-	0.32	MS, RI
88.	Sandaracopimarinal	2157	2185	0.93	-	-	MS, RI
89.	Octadecamethyl-cyclononasiloxane	2198	2200	8.42	0.48	-	MS, RI
90.	Heneicosane	2276	2109	-	-	12.67	MS, RI
91.	Octacosane	2764	2800	-	0.16	-	MS, RI
92.	Squalene	2797	2790	0.50	0.03	0.13	MS, RI
93.	Nonacosane	2856	2900	-	-	5.87	MS, RI
94.	Tetrateracontane	3113	3028	4.30	0.54	4.02	MS, RI
95.	Hexatriacontane	3209	3597	28.44	-	14.50	MS, RI
Monoterpene hydrocarbons				9.01	0.50	-	
Oxygen containing monoterpene				3.13	0.58	1.36	
Sesquiterpene hydrocarbons				0.64	24.55	8.96	
Oxygen containing sesquiterpene				3.53	50.31	4.61	
Fatty acid esters				22.26	-	7.83	
Others				53.04	14.18	63.48	
Total identified components				91.61	90. 12	86.24	

^a^ Arrangement of the compounds based on their elution on RTX-5MS column. ^b^ Kovats index determined experimentally on RTX-5MS column relative to a standard mixture of C8–C30 n-alkanes. ^c^ Published Kovats retention indices. Identification was based on comparison of the compounds mass spectral data (MS) and Kovats retention indices (RI) with those of NIST Mass Spectral Library (2011), Wiley Registry of Mass Spectral Data 8th edition and literature.

**Table 2:** Mean biofilm inhibitory activity (µg/mL) of *J. intigrimma*, *J. gossypifolia* and *J. roseae* essential oils against *Escherichia coli* determined by modified method of biofilm inhibition spectrophotometric assay.

Sample Conc. (µg/mL)	Mean Biofilm Inhibitory Activity %		
	*J. intigrimma*	*J. gossypifolia*	*J. roseae*
7.81	52.14 ± 1.3	0	0
15.63	76.38 ± 2.5	0	16.31 ± 1.9
31.25	100 ± 0	0	38.82 ± 1.3
62.5	100 ± 0	0	62.25 ± 2.5
125	100 ± 0	5.08 ± 2.1	76.35 ± 0.72
250	100 ± 0	17.36 ± 1.5	100 ± 0
500	100 ± 0	28.14 ± 1.2	100 ± 0
1000	100 ± 0	39.25 ± 0.58	100 ± 0
MIC	31.25	>1000	250

Data are presented as means ± S.D. *n* = 3.

**Table 3:** Free binding energies (kcal/mol) of major compounds in the active site of *E. coli* adhesion protein FimH using *in silico* studies.

Compound	Adhesion Protein FimH (1TR7)	Number of Formed Hydrogen Bonds	Number of Formed Alkyl and π-Alkyl Bonds
D-Limonene	21.10	-	3; Ile52, Ile13
Isosativene	52.27	-	3; Ile52, Ile13, Tyr48
*α*-Copaene	4.05	-	7; Ile52, Ile13, Tyr137, Tyr48
Silphiperfol-6-ene	52.27		9; Ile52, Ile13, Tyr137, Phe142
*α*-Guaiene	44.19	-	5; Ile52, Ile13, Phe142, Tyr48
*β*-Caryophyllene	17.04	-	4; Ile52, Ile13, Tyr48
Germacrene D	8.63	-	3; Phe142, Ile13, Tyr48
*β-*Ionone	10.36	1; Phe1	1; Ile13
Cubebol	−8.92	1; Asp140	2; Phe142, Ile13
δ-Cadinene	44.02	-	4; Ile52, Ile13, Tyr137
Caryophyllene oxide	0.82	1; Phe1	4; Phe142, Ile13, Tyr137
Spathulenol	47.82	1; Phe1	7; Ile52, Ile13, Tyr137, Tyr48, Phe142
Copaborneol	49.78	1; Asp140	4; Ile52, Ile13, Tyr137, Tyr48
Muurola-4,10(14)-dien-1β-ol	29.16	1; Asp140	6; Phe142, Ile13, Tyr137, Tyr48
Eudesma-4(15),7-dien-1*β*-ol	27.03	1; Phe1	6; Phe142, Ile13, Ile52, Tyr48
Caryophylla-4(12),8(13)-dien-5*α*-ol	12.29	2; Asp54, Phe1	3; Phe142, Ile13,
Methyl linoleate	−4.55	2; Asn135, Phe1	1; Ile52
7,10-Hexadecadienoic acid, methyl ester	−6.70	1; Phe1	1; Ile52
Geranyl linalool	42.39	1; Phe1	2; Tyr48, Tyr137
9,12,15-Octadecatrienoic acid, methyl ester	8.86	2; Asp47, Phe1	1; Ile52
Heneicosane	−30.68	-	2; Ile52, Tyr48
Nonacosane	FD	-	-
Tetrateracontane	FD	-	-
Hexatriacontane	FD	-	-

Positive values indicate unfavorable interaction. FD: fail to dock.

## Data Availability

Data are available in the manuscript.

## References

[1] Youssef F.S., Hamoud R., Ashour M.L., Singab A.N., Wink M. (2014). Volatile oils from the aerial parts of *Eremophila maculata* and their antimicrobial activity. Chem. Biodivers..

[2] Mokni R.E., Youssef F.S., Jmii H., Khmiri A., Bouazzi S., Jlassi I., Jaidane H., Dhaouadi H., Ashour M.L., Hammami S. (2019). The Essential oil of Tunisian *Dysphania ambrosioides* and its antimicrobial and antiviral properties. J. Ess.Oil Bear. Plants.

[3] Gamal El-Din M.I., Youssef F.S., Ashour M.L., Eldahshan O.A., Singab A.N.B. (2018). Comparative analysis of volatile constituents of *Pachira aquatica* Aubl. and Pachira glabra Pasq., their anti-Mycobacterial and anti-Helicobacter pylori activities and their metabolic discrimination using chemometrics. J. Ess.Oil Bear. Plants Bear. Plants.

[4] Bakkali F., Averbeck S., Averbeck D., Idaomar M. (2008). Biological effects of essential oils—A review. Food Chem. Toxicol..

[5] Altyar A.E., Ashour M.L., Youssef F.S. (2020). *Premna odorata*: Seasonal metabolic variation in the essential oil composition of its leaf and verification of its anti-ageing potential via *in vitro* assays and molecular Modelling. Biomolecules.

[6] Jamal M., Ahmad W., Andleeb S., Jalil F., Imran M., Nawaz M.A., Hussain T., Ali M., Rafiq M., Kamil M.A. (2018). Bacterial biofilm and associated infections. J. Chin. Med.Assoc..

[7] Hurlow J., Couch K., Laforet K., Bolton L., Metcalf D., Bowler P. (2015). Clinical biofilms: A challenging frontier in wound care. Adv. Wound Care..

[8] Sutherland I.W. (2001). The biofilm matrix–an immobilized but dynamic microbial environment. Trends Microbiol..

[9] Salmani A., Shakerimoghaddam A., Pirouzi A., Delkhosh Y., Eshraghi M. (2020). Correlation between biofilm formation and antibiotic susceptibility pattern in Acinetobacter baumannii MDR isolates retrieved from burn patients. Gene Rep..

[10] Devappa R.K., Makkar H.P., Becker K. (2011). Jatropha diterpenes: A review. J. Am. Oil Chem. Soci..

[11] Okoh S.O., Iweriebor B.C., Okoh O.O., Nwodo U.U., Okoh A.I. (2016). Antibacterial and antioxidant properties of the leaves and stem essential oils of *Jatropha gossypifolia* L.. BioMed Res. Int..

[12] Eshilokun A.O., Kasali A.A., Ogunwande I.A., Walker T.M., Setzer W.N. (2007). Chemical composition and antimicrobial studies of the essential oils of *Jatropha integerrima* Jacq (leaf and seeds). Nat. Prod. Commun..

[13] Youssef F.S., Mamatkhanova M.A., Mamadalieva N.Z., Zengin G., Aripova S.F., Alshammari E., Ashour M.L. (2020). Chemical profiling and discrimination of essential oils from six Ferula species using GC analyses coupled with chemometrics and evaluation of their antioxidant and enzyme inhibitory potential. Antibiotics.

[14] Aboaba S.A., Adebayo M.A., Ogunwande I.A., Olayiwola T.O. (2015). Volatile constituents of *Jatropha gossypifolia* L. grown in Nigeria. Am. J. Ess. Oils Nat. Prod..

[15] Barra A. (2009). Factors affecting chemical variability of essential oils: A review of recent developments. Nat. Prod. Comm..

[16] Hammer K.A., Carson C.F., Riley T.V. (1999). Antimicrobial activity of essential oils and other plant extracts. J. Appl. Microbiol..

[17] Ghaneian M.T., Ehrampoush M.H., Jebali A., Hekmatimoghaddam S., Mahmoudi M. (2015). Antimicrobial activity, toxicity and stability of phytol as a novel surface disinfectant. Environ. Health Eng. Manag. J..

[18] Islam M.T., Ali E.S., Uddin S.J., Shaw S., Islam M.A., Ahmed M.I., Shill M.C., Karmakar U.K., Yarla N.S., Khan I.N. (2018). Phytol: A review of biomedical activities. Food Chem. Toxicol..

[19] Schmidt E., Bail S., Friedl S.M., Jirovetz L., Buchbauer G., Wanner J., Denkova Z., Slavchev A., Stoyanova A., Geissler M. (2010). Antimicrobial activities of single aroma compounds. Nat. Prod. Commun..

[20] Donlan R.M. (2002). Biofilms: Microbial life on surfaces. Emerg. Infect. Dis..

[21] Klemm P., Vejborg R.M., Hancock V. (2010). Prevention of bacterial adhesion. App. Microbiol. Biotechnol..

[22] Cerca N., Martins S., Pier G.B., Oliveira R., Azeredo J. (2005). The relationship between inhibition of bacterial adhesion to a solid surface by sub-MICs of antibiotics and subsequent development of a biofilm. Res. Microbiol..

[23] Lee J.H., Kim Y.G., Khadke S.K., Lee J. (2021). Antibiofilm and antifungal activities of medium-chain fatty acids against *Candida albicans via* mimicking of the quorum-sensing molecule farnesol. Microb. Biotechnol..

[24] Vijay K., Kiran G.S., Divya S., Thangavel K., Thangavelu S., Dhandapani R., Selvin J. (2021). Fatty acid methyl esters from the coral-associated bacterium *Pseudomonas aeruginosa* inhibit virulence and biofilm phenotypes in multidrug resistant *Staphylococcus aureus*: An *in vitro* approach. Front. Microbiol..

[25] Da Silva E.G., Bandeira Junior G., Cargnelutti J.F., Santos R.C.V., Gündel A., Baldisserotto B. (2021). *In vitro* antimicrobial and antibiofilm activity of S-(-)-limonene and R-(+)-limonene against fish bacteria. Fishes.

[26] Mishra R., Panda A.K., De Mandal S., Shakeel M., Bisht S.S., Khan J. (2020). Natural anti-biofilm agents: Strategies to control biofilm-forming pathogens. Front. Microbiol..

[27] Ayoub I.M., Youssef F.S., El-Shazly M., Ashour M.L., Singab A.N.B., Wink M. (2015). Volatile constituents of *Dietes bicolor* (Iridaceae) and their antimicrobial activity. Z. Naturforsch. C.

[28] Mamadalieva N.Z., Youssef F.S., Ashour M.L., Sasmakov S.A., Tiezzi A., Azimova S.S. (2019). Chemical composition, antimicrobial and antioxidant activities of the essential oils of three Uzbek Lamiaceae species. Nat. Prod. Res..

[29] Youssef F.S., Ovidi E., Musayeib N.M.A., Ashour M.L. (2021). Morphology, anatomy and secondary metabolites investigations of *Premna odorata* Blanco and evaluation of its anti-tuberculosis activity using *in vitro* and *in silico* studies. Plants.

[30] Kiehlbauch J.A., Hannett G.E., Salfinger M., Archinal W., Monserrat C., Carlyn C. (2000). Use of the National Committee for Clinical Laboratory Standards guidelines for disk diffusion susceptibility testing in New York state laboratories. J. Clin. Microbiol..

[31] Hindler J., Howard B., Keiser J., Howard B.J. (1994). Antimicrobial agents and antimicrobial susceptibility testing. Clinical Pathogenic Microbiology.

[32] Regev-Shoshani G., Ko M., Miller C., Av-Gay Y. (2010). Slow release of nitric oxide from charged catheters and its effect on biofilm formation by *Escherichia coli*. Antimicrob. Agents Chemother..

[33] Adnan M., Patel M., Deshpande S., Alreshidi M., Siddiqui A.J., Reddy M.N., Emira N., De Feo V. (2020). Effect of Adiantum philippense extract on biofilm formation, adhesion with its antibacterial activities against foodborne pathogens, and characterization of bioactive metabolites: An *in vitro*-*in silico* approach. Front. Microbiol..

[34] Thabet A.A., Youssef F.S., El-Shazly M., El-Beshbishy H.A., Singab A.N.B. (2018). Validation of the antihyperglycaemic and hepatoprotective activity of the flavonoid rich fraction of *Brachychiton rupestris* using in vivo experimental models and molecular modelling. Food Chem. Toxicol..

[35] Talaat A.N., Ebada S.S., Labib R.M., Esmat A., Youssef F.S., Singab A.N.B. (2018). Verification of the anti-inflammatory activity of the polyphenolic-rich fraction of *Araucaria bidwillii* Hook. using phytohaemagglutinin-stimulated human peripheral blood mononuclear cells and virtual screening.. J. Ethnopharmacol..

[36] Labib R., Youssef F., Ashour M., Abdel-Daim M., Ross S. (2017). Chemical composition of *Pinus roxburghii* Bark volatile oil and validation of its anti-inflammatory activity using molecular modelling and bleomycin-induced inflammation in albino Mice. Molecules.

